# Comparison of methanol and isopropanol as wash solvents for determination of hair cortisol concentration in grizzly bears and polar bears

**DOI:** 10.1016/j.mex.2017.01.004

**Published:** 2017-01-30

**Authors:** Thomas Kroshko, Luciene Kapronczai, Marc R.L. Cattet, Bryan J. Macbeth, Gordon B. Stenhouse, Martyn E. Obbard, David M. Janz

**Affiliations:** a Toxicology Centre, University of Saskatchewan, Saskatoon, SK, S7N 5B3, Canada; b RGL Recovery Wildlife Health & Veterinary Services, Saskatoon, SK, S7H 4A6, Canada; c Department of Veterinary Pathology, University of Saskatchewan, Saskatoon, SK, S7N 5B4, Canada; d Department of Ecosystem and Public Health, University of Calgary, Calgary, AB, T2N 4Z6, Canada; e Foothills Research Institute, Hinton, AB, T7V 1X6, Canada; f Wildlife Research and Monitoring Section, Ontario Ministry of Natural Resources and Forestry, Trent University, Peterborough, ON, K9J 7B8, Canada; g Department of Veterinary Biomedical Sciences, University of Saskatchewan, 52 Campus Drive, Saskatoon, SK, S7N 5B4, Canada

**Keywords:** Hair cortisol concentration, Hair cortisol, HCC, Hair analysis, Long-term stress, Wash solvent, Wildlife, Bear

## Abstract

Methodological differences among laboratories are recognized as significant sources of variation in quantification of hair cortisol concentration (HCC). An important step in processing hair, particularly when collected from wildlife, is the choice of solvent used to remove or “wash” external hair shaft cortisol prior to quantification of HCC. The present study systematically compared methanol and isopropanol as wash solvents for their efficiency at removing external cortisol without extracting internal hair shaft cortisol in samples collected from free-ranging grizzly bears and polar bears. Cortisol concentrations in solvents and hair were determined in each of one to eight washes of hair with each solvent independently.

• There were no significant decreases in internal hair shaft cortisol among all eight washes for either solvent, although methanol removed detectable hair surface cortisol after one wash in grizzly bear hair whereas hair surface cortisol was detected in all eight isopropanol washes.
• There were no significant differences in polar bear HCC washed one to eight times with either solvent, but grizzly bear HCC was significantly greater in hair washed with isopropanol compared to methanol.
• There were significant differences in HCC quantified using different commercial ELISA kits commonly used for HCC determinations.

## Graphical abstract

**Figure:**
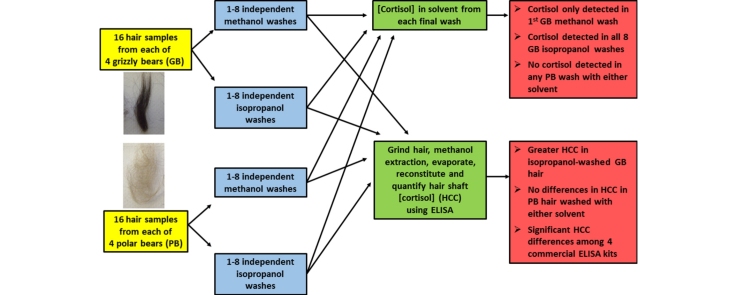

## Method details

### Background

Quantification of hair cortisol concentration (HCC) has emerged as a promising tool to investigate long-term stress in mammals. Since the first report of hair cortisol in wildlife [1], there are now many publications that have used this approach in wildlife, veterinary, zoo, and human research [2], [3]. However, methodologies used for sample collection, storage, preparation and cortisol quantification vary considerably, and recent papers have identified concerns about the reproducibility of HCC determinations [2], [3], [4], [5], [6]. One of these concerns is the presence of cortisol on the exterior of the hair shaft and how best to remove it [3], [7].

Given the certainty that cortisol concentrations in blood and sweat are greater than that found within the hair shaft, it is clear that not removing it will cause artefactual increases in HCC [3]. This is especially critical when measuring HCC in free-ranging wildlife such as bears, because hair is commonly contaminated with blood, feces, urine and lipids [8], [9], [10]. Intact hair is typically washed with isopropanol [7], [9], [10] or methanol [8], [11], [12] three to five times in brief (3 min) intervals, depending on the extent of external contamination [8]. Multiple short washes are required to prevent the solvent from penetrating into the hair and removing internal hair shaft (medulla or hair matrix) cortisol, which also complicates the solvent choice because methanol is more effective at removing external cortisol than isopropanol, but also penetrates the hair shaft more readily [13], [14]. Another methodological consideration is the technique used to quantify HCC. Most researchers have adapted enzyme-linked immunosorbent assays (ELISA) designed for saliva (e.g., [7], [9]) or serum (e.g., [8]), although there has been some use of radioimmunoassay and liquid chromatography–tandem mass spectrometry [11], [15], [16]. The main objective of the present study was to compare isopropanol and methanol as wash solvents to remove external hair shaft cortisol contamination, without lowering internal hair shaft cortisol, in grizzly bear (*Ursus arctos*) and polar bear (*Ursus maritimus*) hair. A secondary objective was to compare the HCC results obtained from commonly-used commercial cortisol ELISA kits.

### Hair sample preparation and analysis – solvent wash dynamics

Guard hair samples from four grizzly bears and four polar bears were collected during previous studies and stored in paper envelopes in the dark at room temperature [8], [17]. Three of the grizzly bear hair samples were collected from bears captured using leg snares in 2007 or 2008, following an approved animal use protocol (AUP 20010016) by the University of Saskatchewan Committee on Animal Care and Supply. One of the grizzly bear hair samples was collected opportunistically in 2007 from a bear killed in self-defence. All grizzly bear samples were collected from animals inhabiting the eastern foothills of the Rocky Mountains near Hinton, AB, Canada. Polar bear hair samples were collected in 2008 from the southern Hudson Bay subpopulation (ON, Canada). Polar bears were captured using helicopter darting, following an approved protocol by the Animal Care Committee of the Ontario Ministry of Natural Resources and Forestry (permit 08-95).

From each bear, 16 guard hair samples of approximately 50 mg were collected, with each 16 sample set being as identical as possible with regard to visual appearance, extent of visible contamination with biologicals and dirt, and body location. Eight of the 16 samples from each bear were used to study methanol wash dynamics, and eight for isopropanol. As described previously [7], [8], a single wash cycle consisted of the hair being mixed with 2.0 ml of the appropriate solvent for 3 min on a slow rotator, after which hair was removed from the solvent and dried thoroughly. Each successive sample from each bear was washed one additional time than the last, starting with one wash and ending with eight. The solvent from each sample’s final wash cycle (i.e., one to eight washes), as well as the washed hair from each wash cycle, were used for cortisol determinations.

The remaining sample preparation and cortisol analysis followed previously validated procedures [8], [17]. Briefly, hair was ground into a fine powder using a Retsch MM 301 Mixer Mill (Retsch Inc., Newtown, PA), and 25 mg of powdered hair was incubated for 12 h on a slow rotator in the presence of 0.5 ml of methanol to extract cortisol. The methanol was separated from the powder by centrifugation for 15 min at 2150*g*, evaporated at 38 °C under nitrogen gas, and reconstituted in 0.2 ml of phosphate buffer (assay diluent buffer from the kit). Cortisol was quantified using a commercially available ELISA kit (catalogue EA65, Oxford Biomedical, Lansing, MI). Each sample’s final solvent wash solution was similarly evaporated, reconstituted in 0.4 ml of buffer, and assayed for cortisol. Performance characteristics of the ELISA in grizzly and polar bears were: intra-assay % coefficients of variation (%CV) of 3.58–9.74%, inter-assay%CVs of 5.75–11.44%, and extraction efficiencies of 94.2 ± 5.1% for grizzly bear and 95.6 ± 3.2% for polar bear. Parallelism between serially diluted hair extracts and the kit standard curve were observed for both bear species. The limit of detection was 0.04 ng/ml, which corresponds to a HCC of 0.32 pg cortisol/mg hair for a 25 mg processed hair sample [8], [17].

### Sample preparation and analysis – cortisol ELISA kit comparison

A total of seven 50 mg hair samples (from four grizzly bears and three polar bears) were used for the ELISA kit comparison. Each hair sample was prepared as described above, except that all received a total of three wash cycles with either methanol or isopropanol. The samples were then ground, extracted, dried as previously described and reconstituted in the appropriate assay diluent buffer provided with each kit. Three commercially available ELISA kits were used to compare mean cortisol values: the Oxford EA65 kit described above (ELISA1), a salivary cortisol ELISA (ELISA2; catalogue 1-3002, Salimetrics LLC, State College, PA), and a cortisol ELISA from Cayman (ELISA3; catalogue 500360, Cayman Chemical, Ann Arbor, MI). In addition, Oxford Biomedical modified their EA65 kit in 2016 with a new primary antibody, and this newer kit was also used for the kit comparison (ELISA4). For statistical comparison among kits, cortisol values were combined for both species and solvents to increase sample size. Cross-reactivity of the primary antibodies used in these kits with related steroids and pharmaceuticals are provided on the company webpages: (http://www.oxfordbiomed.com/sites/default/files/spec_sheet/EA65.pdf; https://www.salimetrics.com/assets/documents/1-3002n.pdf; https://www.caymanchem.com/pdfs/500360.pdf).

### Statistical analyses

Differences among sequential methanol and isopropanol wash data for HCC and solvent cortisol concentrations were detected using repeated measures analysis of variance. Differences between methanol- and isopropanol-washed HCC in grizzly bear and polar bear hair were detected using paired *t*-tests. These analyses were conducted using IBM SPSS Statistics (Version 23; IBM Corporation, Armonk, NY). To compare between HCC quantified using different ELISA kits, we used the ‘glmer’ function in package ‘lme4’ [18], and the ‘glht’ function in package ‘multcomp’ [19], in R 3.2.4 [20] to model the data using generalized linear mixed effects and to simultaneously compare among multiple means. An α value of 0.05 was used for statistical significance. Data were expressed as mean ± standard deviation or mean ± 95% confidence interval, as indicated in the text.

#### Repeated solvent wash cycles

For grizzly bears, no significant difference in HCC was detected among one through eight independent washes with methanol (F(7,21) = 2.43, *p* = 0.054, *n* = 32), although there was a trend for lesser HCC in the samples washed seven and eight times with methanol despite no corresponding appearance of cortisol in the washes (Table 1). A significant difference was observed among HCC values when grizzly bear hair was washed with isopropanol (F(7,21) = 2.97, *p* = 0.025, *n* = 32), although there was no decreasing trend observed in the data (Table 1). Analysis of the final methanol aliquot used in washing each grizzly bear hair sample detected cortisol in the first wash, with the rest of the washes falling below detection limit (Table 1). In contrast, cortisol was detected in all 8 isopropanol washes of grizzly bear hair (Table 1). However this does not appear to be internal hair shaft cortisol, since concurrent quantification of HCC in the same samples were similar among all isopropanol washes, ranging from 1.15 to 1.49 pg/mg with no decreasing trend as number of washes progressed. A possible explanation is that isopropanol, but not methanol, may extract substance(s) from the external surface of grizzly bear hair that non-specifically cross-react with the primary antibody used in this ELISA. Interestingly, this was not observed for polar bear hair washed up to eight times with isopropanol, where all wash solvent cortisol concentrations were below the detection limit. It was not feasible due to funding constraints in the present study to determine whether the reconstituted isopropanol washes from grizzly bears had detectable cortisol using other cortisol ELISAs with different primary antibodies.

**Table 1:** Cortisol concentrations in grizzly bear and polar bear hair, and grizzly bear wash solvents, following 1–8 independent, sequential washes with either methanol or isopropanol. Results for polar bear wash solvents are not presented because all samples had cortisol concentrations below the detection limit (0.04 ng/ml) of the assay. The *p* value indicates statistical significance among washes determined using repeated measures analysis of variance. Data are mean ± standard deviation. ND: not detected. N/A: not applicable.

	Grizzly Bear Hair (pg/mg)		Grizzly Bear Wash (pg/ml)		Polar Bear Hair (pg/mg)	
Wash	Methanol	Isopropanol	Methanol	Isopropanol	Methanol	Isopropanol
1	0.85 ± 0.45	1.15 ± 0.54	4.68 ± 3.72	14.58 ± 3.53	0.42 ± 0.24	0.43 ± 0.37
2	0.77 ± 0.39	1.26 ± 0.65	ND	12.06 ± 3.82	0.47 ± 0.34	0.47 ± 0.35
3	0.88 ± 0.33	1.25 ± 0.55	ND	11.62 ± 3.13	0.54 ± 0.36	0.49 ± 0.32
4	0.97 ± 0.57	1.40 ± 0.66	ND	15.27 ± 4.47	0.54 ± 0.30	0.53 ± 0.42
5	0.67 ± 0.38	1.49 ± 0.55	ND	10.40 ± 3.53	0.48 ± 0.29	0.55 ± 0.43
6	0.87 ± 0.38	1.32 ± 0.63	ND	8.75 ± 6.61	0.51 ± 0.34	0.58 ± 0.43
7	0.74 ± 0.45	1.21 ± 0.51	ND	10.11 ± 1.80	0.50 ± 0.37	0.47 ± 0.31
8	0.59 ± 0.09	1.16 ± 0.50	ND	8.74 ± 0.99	0.47 ± 0.27	0.40 ± 0.25
*p* value	0.054	0.025	N/A	0.001	0.41	0.086

There were no differences observed in HCC of polar bear hair washed 1 through 8 times with methanol (F(7,21) = 1.084, *p* = 0.408, *n* = 32) or isopropanol (F(7,21) = 2.125, *p* = 0.086, *n* = 32; Table 1). No analyses could be carried out on the isopropanol or methanol wash series for polar bears because all samples had cortisol concentrations below the detection limit (0.04 ng/ml) of the assay. Previous research with externally contaminated grizzly bear and polar bear hair reported that cortisol concentrations in the first two methanol washes were between 10 and 100 fold greater than hair shaft cortisol concentrations [8], [21]. Similarly greater wash cortisol concentrations in each of three consecutive isopropanol washes of polar bear hair contaminated with blood or lipids were previously reported [9]. Although there was a trend for decreasing HCC in grizzly bear hair washed seven or eight times, there was no corresponding cortisol detected in the wash solvent. Previous validation work with polar bear hair detected cortisol in the sixth and seventh washes in three of 18 bears studied [21]. Thus, for contaminated hair samples collected from free-ranging wildlife such as bears, it is recommended that three to four brief (3 min) methanol washes be performed, depending on the degree of contamination [8], [21]. Using this approach, this more aggressive solvent was previously reported to remove all detectable external hair cortisol from even heavily contaminated grizzly bear [8] and polar bear [17] samples. The present study confirms that this procedure will not extract internal hair shaft cortisol in these species, although further validation is required in other mammals due to potential differences in porosity of the hair cuticle, among other factors.

#### HCC in methanol vs. isopropanol washed hair

**Fig. 1:**
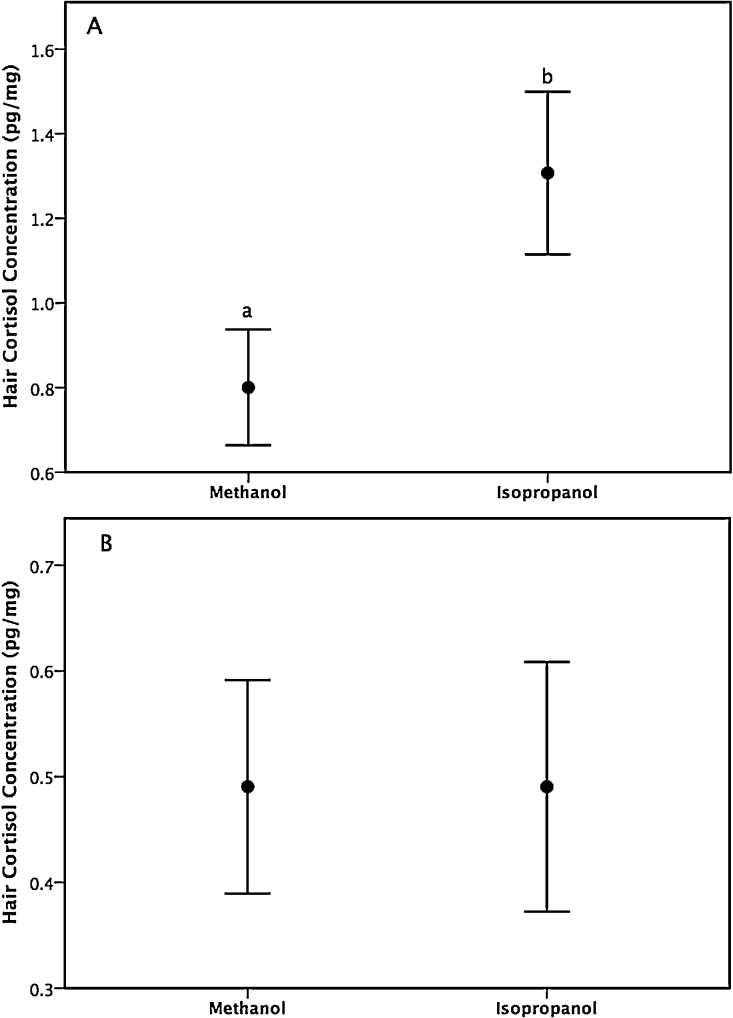
Hair cortisol concentrations in (A) grizzly bear and (B) polar bear hair washed one to eight times with either methanol or isopropanol. Different lower case letters indicate a significant difference as determined using a paired *t*-test (*p* < 0.001). Data are mean ± 95% confidence interval.

Significantly greater HCC values were observed in isopropanol-washed grizzly bear hair (mean ± SD: 1.28 ± 0.517 pg/mg) than in hair samples washed with methanol (0.790 ± 0.371 pg/mg; *t*(31) = −9.94, *p <* 0.001; Fig. 1A). No difference was detected in HCC of polar bear hair washed in isopropanol (0.491 ± 0.328 pg/mg) compared to hair washed in methanol (0.491 ± 0.280 pg/mg; *t*(31) = 0.002, *p* = 0.998; Fig. 1B). Although grizzly bear hair washed with methanol contained lesser HCC than hair washed with isopropanol, repeated wash cycles with methanol did not result in lower HCC being quantified in grizzly bears, and this difference was not seen in polar bears. In addition, no difference was observed in polar bear hair washed with methanol compared with isopropanol, which serves as further evidence that methanol is not extracting internal hair shaft cortisol.

#### Comparison of HCC using different ELISA kits

**Fig. 2:**
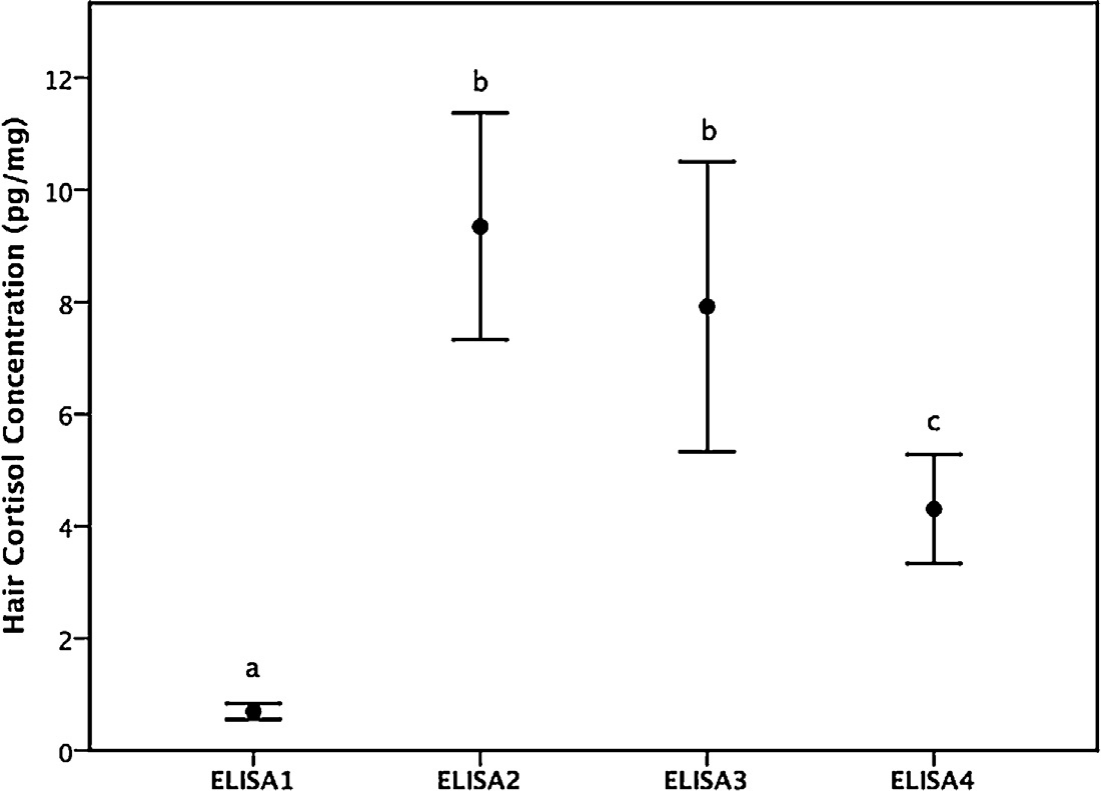
Hair cortisol concentrations quantified using different commercial ELISA kits (see text for description). Different lower case letters indicate significant differences as determined using simultaneous multiple comparisons of means by Tukey contrasts. Data are mean ± 95% confidence interval.

Grizzly and polar bear hair washed three times with either methanol or isopropanol was assayed for HCC using commercial ELISA kits described above, and HCC data from both species and solvents were combined for simultaneous multiple comparisons of HCC mean values obtained from each kit. The means were calculated using a generalized mixed-effects model (gamma distribution with an inverse link) with HCC as the response variable, ELISA kit as the sole factor, and bear identification as a random effect. ELISA1 provided lower HCC values (mean ± SD: 0.70 ± 0.27 pg/mg) than ELISA2 (9.35 ± 3.80 pg/mg), ELISA3 (7.92 ± 4.85 pg/mg) and ELISA4 (4.31 ± 1.69 pg/mg; all *p <* 0.001; Fig. 2). Mean HCC determined using ELISA4 was significantly lesser than HCC determined using ELISA2 (*p <* 0.001) and ELISA3 (*p <* 0.001; Fig. 2). There was no difference in HCC between ELISA2 and ELISA3 (*p* = 0.567).

Although commercial ELISA kits are designed to accurately measure all hormone present in a sample, differences are commonly observed in the results provided by different kits (e.g., [22], [23]). Part of the impetus for the present study arose from approximately 10-fold differences in HCC observed in polar bears from Greenland ([9]; using isopropanol washes and ELISA2) compared to Ontario, Canada ([17]; using methanol washes and ELISA1). Thus, we compared results obtained from these and other commercial ELISA kits commonly used by researchers quantifying HCC. We observed up to a 14-fold difference between kits, with only ELISA2 and ELISA3 providing comparable HCC values. Because all commercial ELISA kits are designed for diagnostic use in serum or saliva, adapting them for quantification of HCC introduces potential for non-specific binding of unique substances extracted from hair that would not be present in the intended media used for analysis. Thus, comparisons of absolute HCC values among studies of the same wildlife species that use different ELISAs should be assessed with caution, although relative differences in HCC among study sites and life history variables of a given species should remain valid.

## Additional information

Quantification of cortisol in various biological matrices has long been used as a measure of hypothalamus-pituitary-adrenal (HPA) axis activation in vertebrate animals subjected to physiological or psychosocial stressors. Acute activation of the HPA axis is an adaptive response to immediate stressors and is routinely detected using matrices such as blood serum/plasma, saliva or sweat (minutes to hours), and urine or feces (hours to days). However, long term (weeks to months) HPA activation is often associated with adverse health outcomes such as reduced growth, immunosuppression, and impaired reproductive capacity. Due to this, there is a recognized need to develop techniques that can reliably determine long term stress in vertebrates, including free-ranging and captive wildlife species, domesticated animals, and humans. Quantification of cortisol within the hair shaft medulla has emerged as a technique to address this need. Similar to lipid soluble drugs and toxicants, free (unbound) cortisol passively diffuses from the bloodstream into the hair shaft during the active period of hair growth [24], and thus represents a chronological record of HPA activation during the active hair growth phase, which lasts for several months to years in most mammals [2], [3].

Effectively removing external hair cortisol while not altering internal hair shaft cortisol is a critical procedural step in the growing use of HCC as a marker of long-term stress in vertebrate animals [7], [13]. This is especially true in wildlife species, where hair can be significantly contaminated with blood, feces, urine and other biologicals prior to HCC analysis. The choice of solvent used for removing external hair contaminants stems mainly from forensic studies detecting banned substances in athletes, such as anabolic steroids and corticosteroids [25], [26]. These procedures generally use stringent washing procedures to avoid false positive results, and include successive washes with solvents such as dichloromethane, aqueous sodium dodecyl sulfate solutions, phosphate buffer, and/or water. An early study evaluating appropriate wash solvents rejected these approaches due to inconsistency when quantifying HCC [7], and today isopropanol or methanol are the main solvents used for HCC analysis. To our knowledge, only one previous study [7] compared methanol and isopropanol as wash solvents for hair prior to HCC determinations, and provided inconclusive results as to whether either solvent removed internal hair shaft cortisol. Steroids such as cortisol and corticosterone are more soluble in lower alcohols such as methanol when compared to alcohols of higher molecular weight such as isopropanol [13]. However, lower alcohols also penetrate the hair shaft more readily and pose a greater risk of extracting internal hair shaft cortisol [14]. Aqueous washing media such as water or phosphate buffer, used by certain laboratories determining HCC, are even more problematic due to greater penetration of the hair shaft than even lower alcohols [14].
